# The Effect of Green Tea Extract on Oxidative Stress and Spatial Learning in Streptozotocin-diabetic Rats

**Published:** 2017

**Authors:** Mohammad Sharifzadeh, Akram Ranjbar, Asieh Hosseini, Mahnaze Khanavi

**Affiliations:** a *Department of Pharmacology and Toxicology, Faculty of Pharmacy, Tehran University of Medical Sciences, Tehran, Iran. *; b *Department of Toxicology and Pharmacology, School of Pharmacy, Hamadan University of Medical Sciences, Hamadan, Iran.*; c *Razi Drug Research Center, Iran University of Medical Sciences, Tehran, Iran. *; d *Department of Pharmacogenosy, Faculty of Pharmacy, Tehran University of Medical Sciences, Tehran, Iran.*

**Keywords:** Spatial memory, Green tea, Oxidative stress, Diabetes, Learning

## Abstract

Diabetes mellitus is associated with distribution of cognitive functioning. Hyperglycemia induced oxidative stress has been proposed as a cause of memory complications of diabetes including cognitive impairment. The aim of this study was to examine total green tea extract (TGTE), a potent free radical scavenger against spatial impairment in Streptozotocin-diabetic rats. Eight weeks after diabetes induction, TGTE was administrated throught drinking water 3 mg/L. The learning and memory behavior was evaluated with Morris water maze task in male rats. Then, for estimation of oxidative stress parameters such as lipid peroxidation (LPO), total antioxidant capacity (FRAP) and total thiol groups in blood were measured. The total green tea extract showed improved cognitive impairment in diabetic groups but these changes weren’t significant. There was also significant increase FRAP level and total thiol groups in treated green tea groups vs. control. group. This study demonstrated the effectiveness of TGTE on spatial impairment and oxidative stress induced in diabetes mellitus.

## Introduction

Diabetes mellitus is a chronic metabolic disorder, characterized by disturbed glucose metabolism due to absolute or relative insulin deficiency (1-3). Results from human and animal studies show that free radical mediated oxidative stress plays an important role in septohippocampial dysfunction in diabetes (4, 5).The mechanisms that seem to be involved in the genesis of oxidative stress in diabetes are glucose autoxidation, protein glycation, formation of advanced glycation end products and the polyol pathway (1). The increased production of reactive oxygen species is often associated with compromised natural antioxidant defense systems in diabetic tissues. In response to oxidative stress, antioxidant enzymes are believed to be induced to protect cellular functions which maintain *in-vivo* homeostasis (6). Brain is extremely susceptible to oxidative stress to oxidative damage and accumulative evidence is indicating that free radicals and other reactive oxygen species play an important role in neurodegeneration and association cognitive decline in aging and Alzheimers disease (7-9).

It is well known that both ageing and age associated neurodegenerative disorders such as Alzheimer’s disease (AD) and Parkinson’s disease are associated with varying degrees of cognitive impairment, which can cause significant morbidity (10). Free radicals and the oxidative stress neuronal changes which mediate these behavioral effects (11).

Mild to moderate impairments of cognitive functioning have been reported in patients with diabetes mellitus (DM) (12, 13). Previous evidence suggests that the hyperglycemia is the major “toxic” effect in the development of diabetic end-organ damage to brain in Type I and Type II diabetes (14). Diabetic patients may express cognitive deficits in young adults, but can be quite marked in the elderly (15). Recent epidemiological studies even report relation between diabetes and dementia: cognitive deficits are also reported in animal models of diabetes. Streptozotocin (STZ) diabetic rats develop learning deficits which can be prevented, but not fully reversed, with insulin treatment (16). The pathogenesis of cerebral dysfunction in diabetes has not been fully elucidated (17). It has been therefore proposed that antioxidant drugs could be used to prevent or to treat age-associated cognitive decline an AD (18). 

Green tea* (Camellia sinensis* L.) is a beverage that is popular worldwide and possess many pharmacological effects, such as anti-mutagenic, anti-proliferative, anticarcinogenic properties, and, more important for our purposes, neuroprotective in models of degenerative disorders . These properties are thought to be mediated by the green tea polyphenols (GTP), the four main components of which are (-)-epigallocatechin gallate (EGCG), (-)-epicatechin gallate (ECG), (-)-epigallocatechin (EGC), and (-)-epicatechin (EC) (19-22). 

Green tea derived products are mainly extracts of green tea in liquid or powder form varying in the proportion of polyphenols (45-90%) and caffeine content (0.4-10%) (23, 24). Epidemiologic observation and laboratory studies have indicated that polyphenolic compounds present in the tea may reduce the risk of various illnesses, including cancer, diabet and coronary heart disease (25, 26). The green tea polyphenols have been shown to possess potent antioxidant activity that is several folds higher than that of Vitamin C and E (27). Therefore in the present study we have investigated the effect of total green tea extract (TGTE) on oxidative damage and spatial cognition in streptozotocin-induced diabetic rats.

## Experimental

*Materials and methods*

*Plant Materials*

The leaves of *Camellia sinensis* L. (Theaceae) were purchased from the market in September 2006. Voucher specimen has been deposited at the Herbarium of the Faculty of Pharmacy, Tehran University of Medical Sciences, Tehran, Iran.

*Plant Extraction*

Dried and finely powdered aerial parts (1000 g) were extracted with methanol 80% (3×5 L) at room temperature for 4 weeks. After removal of the solvent in vacuuo at 50 ˚C, the residue (300 g, 30%, w/w) was stored at 4 °C in sealed vials until usage.

*Chemicals and drugs*

Dithiononitrobenzoic acid (DTNB), Tris base, 1,1,3,3′-tetraethoxypropane (MDA), 2-thiobarbituric acid (TBA), trichloroacetic acid (TCA), n-butanol, 2,4,6-tripyridyl-s-triazine (TPTZ), from Merck Chemical Co. (Tehran), Streptozotocin Sigma Chemical Co and Green tea were used in this study.

*Animals and experimental design*

Male albino rats of Wistar strain weighing approximately 250–300 g obtained from from the Pasteur Institute of Iran, were used throughout this study. They were maintained at an ambient temperature of 25 ± 2 ◦C and 12/12 h of light–dark cycle. The experiments were conducted according to the ethical norms approved by Ethics Committee Guidelines.

The experimental animals were divided into four groups; each group contained 8-10 animals: (i) control rats; (ii) green tea treated control rats; (iii) diabetic rats; (iv) green tea treated diabetic rats. Diabetes was induced by a single intraperitoneal injection of streptozotocin (60 mg (kg body weight)−1) dissolved in 0.2 mL of normal saline. Then days after streptozotocin injection, development of diabetes was confirmed from tail vein blood glucose level. Rats with blood glucose levels of 250 mg/dL with glycosuria were considered to be diabetic. After diabetes induction, green tea extract was administered orally through the drinking water for 8 weeks [3 mg /L] (28).

*Behavioral tests*

*Morris water maze (MWM)*

Spatial memory was evaluated with a conventional MWM, commonly adopted for studying cognitive (29). In our studies, 4-day training trials of animals were conducted. Spatial memory retention was tested 48 h after the training. Training of all groups of rats was conducted in the Morris Water Maze task. The water maze was a black circular tank (136 cm in diameter and 60 cm in height). The tank was filled with water (20 ± 2 ºC) to a depth of 25 cm and was located in a room containing several extra maze cues. 

The Plexiglas escape platform used for the spatial task was submerged at a depth of 1 cm from water surface. Rats received one training session consisting of four trials in a day and were tested on four consecutive days. A trial was started by placing the rat in the pool facing the wall in one of the four quadrants delineated by marks at the four cardinal directions. Rats were allowed to swim to the hidden platform and the escape latency (time to find the hidden platform) and path length (distance traveled to the hidden platform) were recorded. If an animal did not escape within 90 sec, it was manually guided to the escape platform by the experimenter. Rats were allowed to rest on the platform for 20 sec between each trial. This procedure was repeated with each rat from starting positions in all four quadrants. 

The submerged platform was located in the same quadrant on every trial. The interval between the last training trial and the first testing trial was 48 h. The testing included 1 block of 4 trials.

*Simple visual task*

On week 8 after STZ-administration, performance on a ‘simple visual task’ was evaluated. In the simple visual task, the performance of the diabetic animals was impaired compared with that of the controls. The difference between the groups was most evident during the first day of this new task. In the diabetic animals, there was a lower number of correct responses and a marked increase in the number of both reference and working memory errors, compared with the longitudinal study. 

*Biochemical analysis*

*Estimation of oxidative stress parameters*

*Assay of cellular lipid peroxidation (LPO)*

For measuring the rate of lipid peroxidation, TBA was used which reacts with lipid peroxide molecules. The plasma samples were mixed with TCA (20%) and the precipitate was dispersed in H_2_SO_4_ (0.05 M). TBA (0.2% in sodium sulfate 2M) was added and heated for 30 min in boiling water bath. TBARS adducts were extracted by n-butanol and the absorbance was measured at 532 nm. This reaction is formed in acidic pH and high temperature and the maximum absorption is a pink complex in 532 nm (30).

*Assay of total antioxidant capacity (TAC)*

It was measured by ferric reducing ability of plasma (FRAP) method. This method is based on the ability of plasma in reducing Fe^3+^ to Fe^2+^ in the presence of TPTZ. The reaction of Fe^2+^ and TPTZ gives a complex with blue color and maximum absorbance in 593 nm (31).

*Assay of total thiol groups (TTG)*

To evaluate the plasma total thiol molecules, DTNB was used as a reagent. DTNB reacts with thiol molecules and create a yellow complex which has good absorbance at 412 nm in spectrophotometer (32).

*Statistical analysis*

Results were expressed as the mean ± SE. for all animals in each group. Statistical analysis was carried out using one-way analysis of variance (ANOVA) followed by post hoc Dunkan test. Statistically significant variations are compared as follows: control versus green tea treated control; control versus diabetic and diabetic versus green tea treated diabetic rats. Results were considered significantly different if p < 0.05

## Results

*Effect of green tea extract on the behavior animal testes*

**Figure 1:**
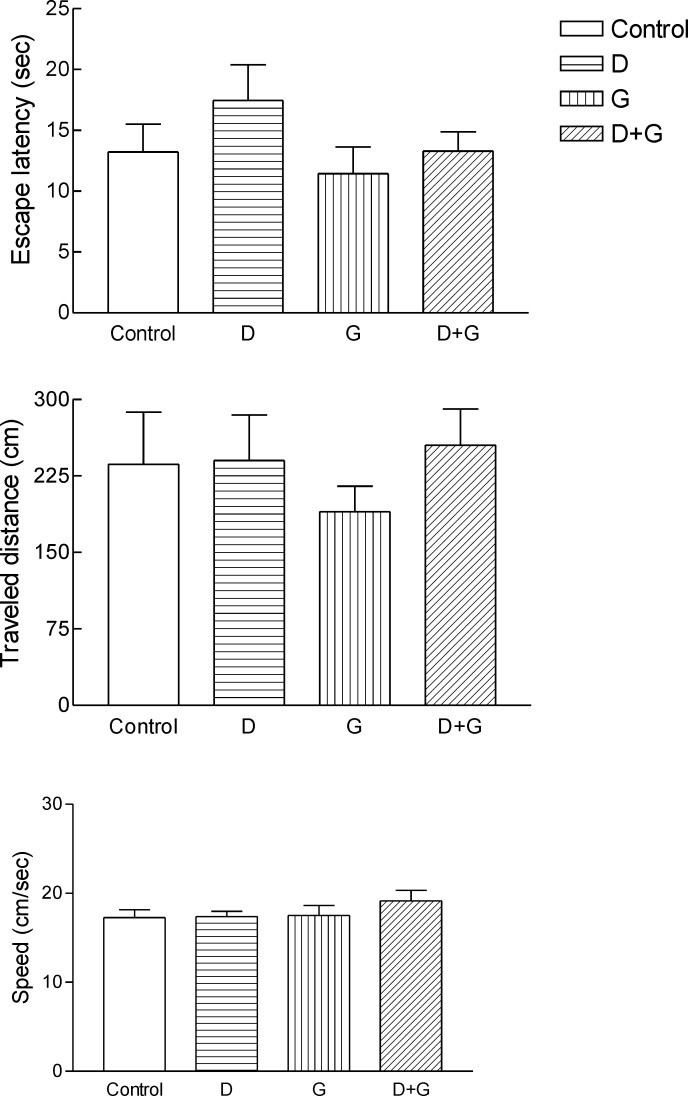
Effect of green tea extracts on the behavior of experimental animals

Administration of GTE decreased escape latency, traveled distance and speed in green tea control and diabetic treated green tea vs. control and diabetic groups. But these changes were not significant (Figure 1.)

*Effect of green tea extract on the oxidative stress parameters and blood glucose levels rats*

Induction of diabetes mellitus was confirmed by blood glucose value above 250 mg/dL. The streptozotocin-induced diabetic rats showed consistent fasting hyperglycemia throughout the study. As shown in result section, green tea treatment to diabetic rats significantly reduced the blood glucose level. Diabetic animals also showed signs of polyuria, polydipsia and polyphagia. 

Result shows the mean ± SD of variables related to either oxidative stress in animals test A significant increase (P = 0.01) in FRAP was observed in green tea vs control and green tea vs. diabetic groups . The values for green tea and control and diabetic groups were 3.66 ± 1.51 and 3.52 ± 0.9 and 1.44 ± 0.62 μmol/mL, respectively. Total thiol groups of diabetic treated with green tea were significantly (P = 0.02) lower than that of green tea controls (0.09 ± 0.04 v.s.0.29 ± 0.1 mM). No significant difference was observed LPO between groups.

## Discussion

The present study demonstrated administration of TGTE improves the performance in water maze tasks and level of parameters oxidative stress such as lipid peroxidation, total antioxidant capacity and total thiol groups in blood correlates with the MWM score. Thus, TGTE may be involved in protecting against neuronal degenerative and accumulation of reactive oxygen species (ROS).In this study, (8 wk) administration of GTE decreased the plasma oxidative status. An increase in the production of free radicals exacerbates the neurodegenerative process by deteriorating cellular enzymes (33). Antioxidative enzymes are activated by GTE intake (34), and the antioxidative potency of human plasma increases with continual ingestion of green tea (35, 36). These antioxidative defense systems might also prevent oxidative damage in the brain. Long-term intake of GTE may be important because cells are constantly exposed to oxidative stress (37).Activated microglia and astrocytes can release cytokines, ROS and nitric oxide (NO) that may contribute to the memory deficits (38, 39).Green tea was reported to reduce the elevated blood glucose level in both type 1 and type 2 diabetic animals (40). Green tea catechins enhance basal and insulin-stimulated glucose uptake (41), inhibit intestinal glucose uptake by sodium-dependent glucose transporter SGLT1 and mimic insulin by decreasing the expression of genes that control gluconeogenesis (42). The administration of green tea caused a significant increase in body weight and reduction in food and water intake in diabetic rats. This could be due to improved glycemic control produced by green tea in diabetic rats(3, 43).

The increase in thiobarbituric acid-reactive substances (TBARS), an index of lipid peroxidation in the diabetic rats might be due to increased levels of oxygen free radicals. In animal studies, tea polyphenol administration was shown to decrease serum TBARS level due to its potential antioxidant activity (34). Green tea can act as scavengers of free radicals. The most essential oxygen-containing free radicals in many disease states are hydroxyl radical, superoxide anion radical, hydrogen peroxide, oxygen singlet, hypochlorite, nitric oxide radical, and peroxynitrite radical. These are highly reactive species, capable in the nucleus, and in the membranes of cells of harmful biologically significant molecules such as DNA, proteins, carbohydrates, and lipids (10). Natural antioxidants can decrease oxidative stress damage induced by a direct scavenging of free radicals (27, 47). Tissue GSH plays a central role in antioxidant defense by detoxifying reactive oxygen species directly or in a glutathione peroxidase catalyzed mechanism. The significant decrease in GSH content may be due to increased utilization of GSH for scavenging effect in diabetes (44). Tea administration was shown to prevent decrease in tissue GSH concentrations in experimental animals (45). Green tea by directly scavenging the free radicals in diabetic rats may reduce the utilization of GSH and thereby exhibiting an increase in the GSH content in green tea treated diabetic rats (46).

**Figure 2.**
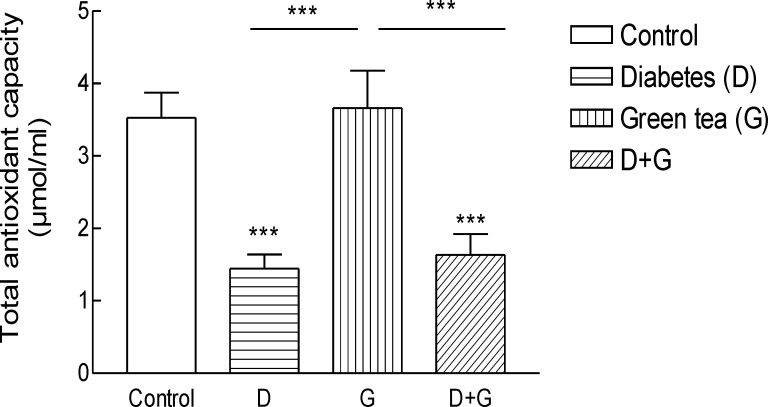
Total antioxidant capacity (TAC) in blood of rats.

**Figure 3:**
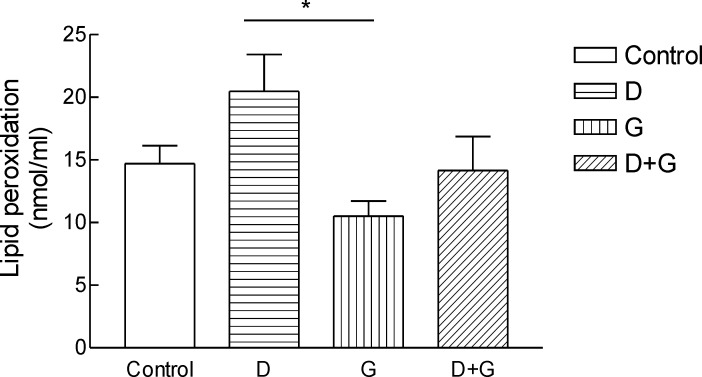
Lipid peroxidation (LPO) in blood of rats. ^***^Significantly different from control and diabetic group at p ‎‎< .05.Groups: Control, D, diabetic; G, green tea; D + G, diabetic +green tea. ‎).

**Figure 4:**
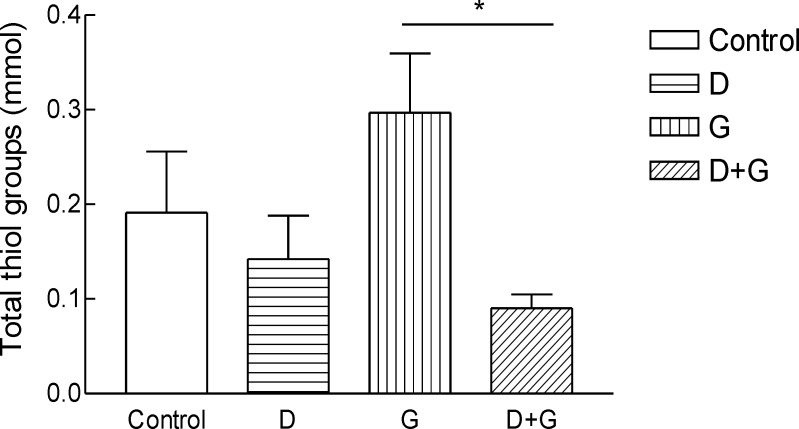
Total thiol groups (TTG) in blood of rats. ^***^Significantly different from control and diabetic group at p ‎‎< .05.Groups: Control, D, diabetic; G, green tea; D + G, diabetic +green tea. ‎).

Apparently, performance deficits in diabetic rats were accentuated by increases in task complexity, and changes in the behavioral protocol. This former finding is in line with an earlier study in which STZ-diabetic mice displayed normal acquisition of relatively simple tasks, but deficits on a more complex task (47, 48). The accentuation of the deficits by changes in the behavioral protocol may reflect an inability of the diabetic rats to adapt their behavioral strategies to the Morris water maze (47). It should also be noted that behavioral deficits in STZ-diabetic rats do not result from direct toxic effects of STZ on the brain (47, 49). Additionally, GTE might be a prophylactic means of preventing neurodegenerative diseases such as Alzheimer’s disease which is associated with oxidative damage and neurotoxicity (50, 51). Long-term administration of an amount of PE that could induce antioxidative effects may protect against age-related declines in memory and learning ability in humans. In the process of aging, LPO accumulates and induces disorders of cellular functions (52, 53). Aging also leads to a decline in spatial memory–related learning ability (54). Therefore, the effects of continued intake of GT_catechin on memory might promote healthy aging of the brain in older (55, 56).There was limitation in the present study such as administration of TGTE in drinking water may be unsuitable and distribution in water wasn’t sufficient for good treatment. Further studies are required to clarify the protective effect of green tea on spatial memory decline and biochemical parameters.
